# Correlation and influencing factors of preoperative anxiety, postoperative pain, and delirium in elderly patients undergoing gastrointestinal cancer surgery

**DOI:** 10.1186/s12871-023-02036-w

**Published:** 2023-03-13

**Authors:** Qing Liu, Liheng Li, Jingwen Wei, Yubo Xie

**Affiliations:** 1 Department of Anesthesiology, The First Affiliated Hospital of Guangxi Medical University, Guangxi, China; 2 Department of Anesthesiology, The Guilin Municipal Hospital of Traditional Chinese Medicine, Guangxi, China; 3 Guangxi Key Laboratory of Enhanced Recovery after Surgery for Gastrointestinal Cancer, The First Affiliated Hospital of Guangxi Medical University, Guangxi, China

**Keywords:** Elderly, Gastrointestinal cancer, Anxiety, Pain, Delirium

## Abstract

**Background:**

The correlation and influencing factors of preoperative anxiety, postoperative pain, and delirium in elderly patients undergoing gastrointestinal cancer surgery were explored with the Beck Anxiety Inventory (BAI) scale, 10-point Visual Analogue Scale (VAS), and Confusion Assessment Method Chinese Reversion (CAM-CR) scale.

**Methods:**

A total of 120 patients aged 65 years old who receiving gastrointestinal cancer surgery were enrolled in the study. Perioperative anxiety, pain, and delirium were assessed by the BAI scale, VAS scale, and CAM-CR scale, respectively. The correlation and influencing factors of preoperative high anxiety, postoperative high pain, and postoperative delirium were analyzed.

**Results:**

Preoperative high anxiety had a moderate positive correlation with postoperative high pain (*P* < 0.001, *r* = 0.410), and had a weak positive correlation with postoperative delirium (*P* = 0.005, *r* = 0.281). postoperative high pain had a weak positive correlation with postoperative delirium (*P* = 0.017, *r* = 0.236). Type of cancer and surgical approach were considered to be independent risk factors of preoperative high anxiety (*P* = 0.006 and *P* = 0.021). Preoperative high anxiety was considered to be an independent risk factor of postoperative high pain (*P*< 0.001). Age and preoperative high anxiety were considered to be independent risk factors of postoperative delirium (*P*< 0.001 and *P* = 0.010).

**Conclusions:**

Elderly patients undergoing gastrointestinal cancer surgery had a higher incidence of preoperative anxiety, as well as first-day postoperative pain and first-day postoperative delirium. Factors such as type of cancer, surgical approach and preoperative anxiety had been identified as influencing preoperative anxiety levels; preoperative anxiety had been linked to postoperative pain; and age and preoperative anxiety have been identified as influencing factors of postoperative delirium.

**Trial registration:**

hiCTR2000032008, 17/04/2020, Title: “Effects of different analgesic methods on postoperative recovery of elderly patients with digestive tract tumor”. Website: https://www.chictr.ogr.cn.

**Supplementary Information:**

The online version contains supplementary material available at 10.1186/s12871-023-02036-w.

## Background

Preoperative anxiety is a typical emotional issue among surgical patients. Studies have shown that preoperative anxiety affects patients on both a physiological and a psychological level [1]. Surgery and anesthesia are the main factors that contribute to preoperative anxiety. The incidence of preoperative anxiety in Chinese adults is 16.9%, and female gender and highly invasive surgery have been identified as risk factors for high preoperative anxiety [2]. Preoperative anxiety has been shown to play an important role in modulating postoperative pain [3–6]. The literature and research are based on the theory of a linear rather than curvilinear relationship between anxiety and pain, meaning that with increased anxiety there is an increase in pain [7]. Earlier psychological intervention for patients with preoperative anxiety have been found to reduce postoperative pain severity [8]. However, Kain et al. [9] pointed out that there was not positive correlation between anxiety and pain, probably due to the correlation between pain and anxiety may not be applicable to patients undergoing major surgery. A study by Kalkman et al. [10] also concluded that preoperative pain was the strongest predictor of severe postoperative pain, rather than preoperative anxiety.

Furthermore, It is still unclear whether preoperative anxiety or postoperative pain contributed to postoperative delirium. Studies have shown that there was no significant correlation between preoperative anxiety and postoperative delirium in aged patients undergoing cardiac surgery and hip fracture patients [11–13]. However, a prospective observational cohort study had indicated that preoperative anxiety was a predictor of postoperative delirium in cancer patients conducted by Saho Wada et al. [14].

Delirium, a severe disorder of attention and cognition, is a common postoperative complication among elderly adults, with an incidence of 15 to 25% following major elective surgeries [15]. Generally, it is caused by multiple factors, such as dementia, depression, and advanced age. While there is no concrete evidence that postoperative pain can lead to delirium, research has shown that high levels of postoperative pain and the use of high opioid doses can increase the risk of developing delirium postoperatively [16]. On the other hand, proper pain management postoperatively can help reduce the incidence of postoperative delirium [17].

In this study, the aim was to explore the correlation and influencing factors of preoperative anxiety, postoperative pain, and delirium in elderly patients undergoing gastrointestinal cancer surgery. To do so, anxiety, pain, and delirium scores were obtained on the day before surgery and on the postopertative day 1, 3, and 7 after surgery.

## Methods

### Study population

This was a single-centered, prospective, and observational study approved by the Medical Ethics Committee of the First Affiliated Hospital of Guangxi Medical University (identifier: NO.2019 (KY-E-115)) and registered at www.chictr.org.cn (ChiCTR2000032008). Patients with gastrointestinal cancer were asked to join our clinical trial before surgery, and all the patients participating in the study signed the informed consent forms. Inclusion criteria were as follows: (1) age not less than 65 years old, (2) American Society of Anesthesiologist physical status class I-III, (3) gastrointestinal cancer surgery under general anesthesia, (4) hospital stay at least senven days after surgery, (5) ability to sign informed consent forms and to read and write, (6) a preoperative Mini-Mental State Examination (MMSE) score of 15 or higher. Exclusion criteria were: patients, with mental illness or history of opioid and alcohol abuse, communication impairment, serious postoperative complications, or American Society of Anesthesiologist physical status class not less than IV, or those transferred to the intensive care unit (ICU) after surgery.

### Study Design

A total of 120 patients who underwent elective radical surgery for gastric cancer or colorectal cancer with general anesthesia were recruited. Anesthesia and postoperative analgesia method could be chosen based on the patient’s current situation and the anesthesiologist’s preference. All patients received general anesthesia, including total intravenous anesthesia or combined intravenous and inhalation anesthesia. Patients were either administered a single bilateral transversus abdominis plane block (TAP) with 0.25% ropivacaine, volume 40 ml before surgery, or received patient-controlled intravenous analgesia (PCIA) pump, or received TAP combined with PCIA (TAP-PCIA) or received patient-controlled epidural analgesia (PCEA) pump, or received oral analgesics in the ward without TAP or any analgesic pump.

### Follow-up, retention, and scales evaluation

In this study, the level and occurrence of anxiety, pain, and delirium were assessed on one day before surgery (day-0) and on the postoperative day (pod) 1, 3, and 7 (pod-1, pod-3, pod-7) with Beck Anxiety Inventory (BAI) scale, 10-point Visual Analogue Scale (VAS), and Confusion Assessment Method Chinese Reversion (CAM-CR) scale, respectively.

The BAI [18] scale consisted of 21 items, each describing a common symptom of anxiety. The respondent was asked to rate how much he or she has been bothered by each symptom a 4-point scale ranging from 0 (Not at all) to 3 (Severely—I could barely stand it). The items were summed to obtain a total score that could range from 0 to 63 (0 = absent, 8–15 = mild, 16–25 = moderate, greater or equal to 26 = severe). Patients were diagnosed with high (moderate to severe) anxiety when BAI score ≥ 16.

The VAS score score was converted to a numerical value between 0 and 10 and to a verbal scale (0 = absent, 1–3 = mild, 4–6 = moderate, 7–9 = intense and 10 = unbearable) [19]. Patients were believed to experience high (moderate to severe) pain when VAS score>3 on any day of day-0, pod-1, pod-3 and pod-7.

In the study, CAM Chinese reversion (CAM-CR) scale was used to screen delirium. As a revision of CAM scale, CAM-CR scale [20] was tailor-made for assessment and diagnosis of delirium in elderly Chinese patients. The sensitivity and specificity of CAM-CR were 0.90 and 0.94, respectively. There were 11 fundamental CAM items in the CAM-CR scale. The criteria for each item was separated into categories based on the severity of patients’ symptoms: 1 = absent, 2 = mild, 3 = moderate, 4 = severe. The items were summed to obtain a total score that could range from 11 to 44. CAM-CR score ≤ 19 suggested that the patient did not have delirium; CAM-CR score of 20–22 suggested that the patient was suspicious for delirium; CAM-CR score>22 suggested the patient was diagnosed delirium. Patient was diagnosed with delirium on any day of day-0, pod-1, pod-3, and pod-7.

All scales were operated by a trained anaesthesiologist through face-to-face assessment during hospital stay. In order to promote follow-up and retention, all scale assessments were conducted in a quiet environment and assessed by the same attending anaesthesiologist. Meanwhile, baseline demographics, such as sex, age, occupation, types of cancer, ASA physical status, duration of anesthesia, duration of surgery, and duration in the postanesthesia care unit (PACU), were collected.

### Statistical analysis

SPSS v.20 ((IBM Corp., Armonk, NY, USA) software was used for complete statistical analysis in the present study. Continuous variables were presented as means and SDs if normally distributed and medians and IQRs if not. Oneway analysis of variance (ANOVA) was used to compared the difference of the pain VAS, BAI, and CAM-CR scores in different period. Levene’s Test was used for equality of variances. LSD (L) test was used for equal square difference and Kruskal-Wallis test if not. The correlation of preoperative high anxiety, postoperative high pain, postoperative delirium and baseline data were analyzed by chi square test and of correlation analysis in the cross table. Continuous correction chi square test would be adopted when the current expected count less than 5. We conducted post hoc analyses for using multivariate linear analysis to find influencing factors of preoperative high anxiety, postoperative high pain, postoperative delirium. A *P* value of less than 0.05 was considered statistically significant. Strength of correlation was interpreted as follows: 0.00–0.19, very weak; 0.20–0.39, weak; 0.40–0.59, moderate; 0.60–0.79, strong; and 0.80–1.00, very strong.

### Sample size calculation

Calculation of sample size was based on the correlation between preoperative high anxiety and postoperative high pain. In the pilot study, the Spearman’s Rank Correlation Coefficient (*r*) was 0.5, the alpha level was set at 0.05, the confidence interval type was two-sided, and the width of confidence interval was 0.35. Consequently, 82 patients were necessary for the study; however, a 20% drop-out rate was accounted for, resulting in at least 99 patients included in the study.

## Result

A total of 120 patients were screened for eligibility and had completed follow-ups on the day-0, pod-1, and pod-3. Four patients declined the BAI scale assessment, and six patients declined the CAM-CR scale assessment on the pod-7. The baseline demographics of the study population were presented in Table 1.

**Table 1:** Baseline demographics of study population

Variable	*n* = 120
Sex, male/female, n(%)	87(72.5%)/33(27.5%)
Age, yr, median (IQR)	69 (66 to 73)
Occupation, farmer/retiree/freelancer, n(%)	49(40.8%) /63(52.5%) /8(6.7%)
Types of cancer, gastric/ colon/rectal, n(%)	45(37.5%)/ 42(35.0%)/ 33(27.5%)
ASA physical status, I/II/III, n(%)	15(12.5%)/36(30.0%)/69(57.5%)
Methods of Anesthesia and analgesia, TAP /PCIA /TAP-PCIA / PCEA/ oral analgesics, n(%)	21(17.5%)/43(35.8%)/18(15.0%)/28(23.3%)/10(8.4%)
Surgical approaches, laparoscopy/ laparotomy, n(%)	95(79.2%)/25(20.8%)
Duration of anesthesia, min, median (IQR)	259 (196 to 357)
Duration of Surgery, min, median (IQR)	214 (151 to 308)
Duration in PACU, min, median (IQR)	87 (66 to 118)

### Comparison of the BAI, VAS, and CAM-CR scores on the day-0, pod-1, pod-3, and pod-7 (Tables 2 and 3)

The BAI scores were significantly lower on pod-7 than on day-0, pod-1, and pod-3 (unadjusted *P* < 0.001, *P* < 0.001, and *P* = 0.001, respectively; adjusted *P* < 0.001, *P* < 0.001, and *P* = 0.005, respectively). Comparatively, The BAI scores were lower on pod-1 and pod-3 than on day-0. (unadjusted *P* = 0.004 and *P* < 0.001; adjusted *P* = 0.239 and *P* < 0.001). Additionally, The BAI scores were lower on pod-3 than on pod-1 ((unadjusted *P* = 0.001, adjusted *P* = 0.006) (Table 2). The incidence of high anxiety (BAI score ≥ 16) occurred on day-0, pod-1, pod-3, and pod-7 were 19.2%, 5.8%, 9.2%, and 0.0%, respectively (Table 3).

**Table 2:** Comparison of the BAI, VAS, and CAM-CR scores during perioperative period

	day-0	pod-1	pod-3	pod-7	*P* value
BAI score	10 (8 to 12)	9 (8 to 10) ^*^	8 (7 to 10) ^*^	7 (4 to 8)^*#★^	0.000
VAS score	0 (0 to 0)	3 (2 to 4)^*^	2 (2 to 3)^*#^	2 (1 to 2)^*#★^	0.000
CAM-CR score	15 (14 to 16)	17 (16 to 20)^*^	17 (15 to 19)^* #^	16 (15 to 18)^* #^	0.000

Data are present as median (IQR)

^*^ *P*<0.05 versus day-0, *P*<0.05 versus pod-1, ^#^^★^ *P* <0.05 versus pod-3

day-0 = one day before surgery, pod-1 = postoperative day 1, pod-3 = postoperative day 3, pod-7 = postoperative day 7

**Table 3:** Incidence of high anxiety, high pain, and delirium during perioperative period

	day-0	pod-1	pod-3	pod-7	
High anxiety, n(%)	23 (19.2%)	7 (5.8%)	11(9.2%)	0 (0.0%)	35^a^
High pain, n(%)	0(0.0%)	50 (41.7%)	18(15.0%)	5(4.2%)	57^b^
Delirium, n(%)	0(0.0%)	29(24.2%)	5 (4.2%)	0(0.0%)	31^c^

day-0 = one day before surgery, pod-1 = postoperative day 1, pod-3 = postoperative day 3, pod-7 = postoperative day 7

High anxiety = BAI score ≥ 16, high pain = VAS score>3, diagnosed delirium = CAM-CR score>22

^a^35 patients experienced high anxiety in the four days, of which 6 patients repeatedly experienced high anxiety

^b^57 patients experienced high pain in the four days, of which 16 patients repeatedly experienced high pain

^c^31 patients were diagnosed as delirium in in the four days, of which 3 patients repeatedly were diagnosed as delirium

The results of the VAS scale demonstrated that the postoperative pain scores gradually decreased over time. The pain VAS scores were signifcantly higher on pod-1, pod-3, and pod-7 than on day-0 (unadjusted *P* < 0.001, *P* < 0.001, and *P* < 0.001, respectively; adjusted *P* < 0.001, *P* < 0.001, and *P* < 0.001, respectively). Additionally, the pain VAS scores were signifcantly higher on pod-1 than on pod-3 and pod-7 (unadjusted *P* = 0.001 and *P* < 0.001; adjusted *P* = 0.006 and *P* < 0.001). Moreover, the pain VAS scores were signifcantly higher on pod-3 than on pod-7 (unadjusted *P* = 0.001; adjusted *P* = 0.004) (Table 2). The incidence of high pain (VAS score>3) occurred on day-0, pod-1, pod-3, and pod-7 were 0.0%, 41.7%, 15.0%, and 4.2%, respectively (Table 3).

The CAM-CR scores were significantly higher on pod-1, pod-3, and pod-7 than on day-0 (unadjusted *P* < 0.001, *P* < 0.001, and *P* < 0.001, respectively; adjusted *P* < 0.001, *P* < 0.001, and *P* < 0.001, respectively). Additionally, the CAM-CR scores were higher on pod-1 than on pod-3 and pod-7 (unadjusted *P* = 0.026 and *P* < 0.001; adjusted *P* = 0.157 and *P* < 0.001). Furthermore, the CAM-CR scores were significantly higher on pod-3 than pod-7 (unadjusted *P* = 0.002 and adjusted *P* = 0.014) (Table 2). The incidence of delirium (CAM-CR score>22) occurred on day-0, pod-1, pod-3, and pod-7 were 0.0%, 24.2%, 4.2%, and 0.0%, respectively (Table 3).

23 (19.2%) patients had experienced preoperative high anxiety (BAI score ≥ 16); 57 (47.5%) patients had experienced postoperative high pain (VAS score>3); and 31(25.8%) patients were diagnosed delirium (CAM-CR score>22)

### The correlation and influencing factors of preoperative high anxiety, postoperative high pain, and postoperative delirium and baseline data (Tables 4 and 5)

The results of cross-table continuous corrected chi-square test and Spearman correlation analysis indicated a significant difference between preoperative high anxiety and age, type of cancer, method of anesthesia and analgesia, and surgical approach (*P* = 0.021, *P*< 0.001, *P* = 0.005, and *P*<0.001, respectively). Although preoperative high anxiety was found to have a positive correlation with age and surgical approach, and have a negative correlation with type of cancer and method of anesthesia and analgesia, the correlations were weak or very weak (*r* = 0.125, *r* = 0.324, *r* = *−* 0.367, and *r* = *−* 0.139, respectively). (Table 4). Multiple linear regression analysis of statistically significant variables (age, type of cancer, method of anesthesia and analgesia, and surgical approach) revealed that type of cancer and surgical approach were independent risk factors of preoperative high anxiety (*P* = 0.006 and *P* = 0.021) (Table 5).

**Table 4:** Chi square test of cross table and spearman correlation analysis

Variable	preoperative high anxiety		postoperative high pain		postoperative delirium	
	*P*	*r*	*P*	*r*	*P*	*r*
Sex	0.542	0.079	0.481	−0.083	0.579	0.051
Age	0.021*	0.125	0.408	0.145	0.000**	0.566
Occupation	0.772	−0.027	0.534	0.091	0.038*	0.181
Type of cancer	0.000**	−0.367	0.004*	−0.260	0.183	−0.049
ASA physical status	0.131	0.115	0.320	0.136	0.183	0.020
Method of Anesthesia and analgesia	0.005*	−0.139	0.000**	−0.120	0.295	−0.085
Surgical approach	0.001*	0.324	0.149	0.152	1.000	0.015
Duration of anesthesia	–	–	0.390	0.271	0.591	0.183
Duration of Surgery	–	–	0.319	0.272	0.375	0.088
Duration in PACU	–	–	0.576	−0.057	0.194	0.099
Preoperative high anxiety	–	–	0.000**	0.410	0.005*	0.281
Postoperative high pain	–	–	–	–	0.017*	0.236

*P<0.05, **P<0.001, *r* = Correlation coefficient

**Table 5:** Multiple linear regression analysis of preoperative high anxiety, postoperative high pain, and postoperative delirium

Dependent variable	independent variable	B	Std error	Beta	t	*P*	VIF	R^2^	adjustd R^2^	F
Preoperative high anxiety	constant	−0.491	0.542	–	− 0.906	0.367	–	0.181	0.152	6.333
	age	0.010	0.007	0.118	1.387	0.168	1.017			
	type of cancer	−0.134	0.048	−0.272	−2.785	0.006*	1.337			
	method of Anesthesia and analgesia	0.000	0.029	0.000	0.001	0.999	1.176			
	surgical approach	0.214	0.091	0.221	2.339	0.021*	1.248			
Postoperative high pain	constant	0.582	0.149	–	3.901	0.000	–	0.182	0.161	8.617
	type of cancer	−0.065	0.060	−0.104	−1.090	0.278	1.297			
	Method of Anesthesia and analgesia	−0.017	0.037	−0.042	−0.460	0.646	1.156			
	Preoperative high anxiety	0.465	0.114	0.366	4.060	0.000**	1.153			
Postoperative delirium	constant	−3.279	0.474	–	−6.917	0.000	–	0.390	0.368	18.354
	age	0.048	0.007	0.525	6.929	0.000**	1.082			
	occupation	0.055	0.054	0.077	10.27	0.307	1.066			
	preoperative high anxiety	0.234	0.090	0.209	2.604	0.010*	1.208			
	postoperative high pain	0.057	0.071	0.064	0.793	0.430	1.228			

B = partial regression coefficient, Std error = standard error, Beta = standardized regression coefficient, t = Students t test; VIF = variance inflation factor, R^2^ = coefficient of determination, F = equality of variances. *P<0.05, **P<0.001

Postoperative high pain was significantly affected by type of cancer, method of anesthesia and analgesia, and preoperative high anxiety (*P* = 0.004, *P*< 0.001, and *P*< 0.001, respectively) (Table 4). Preoperative high anxiety had weak or very weak negative correlations with type of cancer, method of anesthesia and analgesia (*r* = − 0.260 and *r* = *−* 0.120), however, it had a moderate positive correlation with preoperative high anxiety (*r* = 0.410). Multiple linear regression analysis of the statistically significant variables (type of cancer, method of anesthesia and analgesia, and preoperative high anxiety) revealed that preoperative high anxiety was an independent risk factor of postoperative high pain (*P*< 0.001) (Table 5). This meant that preoperative high anxiety had a significant positive effect on postoperative high pain.

Postoperative delirium was found to be significantly associated with age, occupation, preoperative high anxiety and postoperative high pain (*P*< 0.001, *P =* 0.038, *P* = 0.005, and *P* = 0.017, respectively) (Table 4). Preoperative delirium has a moderate positive correlation with age (*r =* 0.566), but had weak or very weak positive correlations with occupation, preoperative high anxiety and postoperative high pain (*r* = 0.181, *r* = 0.281, and *r* = 0.236, respectively). Through multiple linear regression analysis of the statistically significant variables (age, occupation, preoperative high anxiety and postoperative high pain), age and preoperative high anxiety were identified as be independent risk factors of postoperative delirium (*P*< 0.001 and *P* = 0.010) (Table 5).

## Discussion

Most of anxiety during perioperative period occurs before surgery, especially the closer to the surgery date, the more serious the anxiety symptoms. Therefore, in order to obtain the most serious preoperative anxiety symptoms, anxiety was assessed on the day before surgery. In this study, the incidence of high anxiety before surgery was much higher than that after surgery. There are several predispositions for anxiety of surgical patients, including fear of surgery and anesthesia, fear of surgical complications, as well as fear of discomfort and pain during or after surgery. Mild anxiety reflected patient’s normal psychological function and would not be harmful. However, high anxiety may induce a series of adverse effects, including the increase of anesthetic drugs [21, 22], aggravating postoperative pain [6], lowering immunity, and delaying healing [23]. The incidence rate of high anxiety was 19.2% in the study, lower than a cross-sectional study [24] involving 3087 adult patients(40.5%). The obvious difference may be caused by patient’s age, sample size and anxiety scales in the two studies.

In the study, the patients experienced high pain mainly on the first day after operation, and the incidence rate was as high as 47.5%. Preoperative high anxiety not only had a moderate positive correlation with postoperative high pain, but also was considered to be an independent risk factor of postoperative high pain. The important role of anxiety in the perioperative period has been recognized for a long time. Most of the available evidences have revealed a positive correlation between preoperative anxiety and postoperative pain [5, 25, 10]. Preoperative anxiety has been considered as an important predictor of postoperative pain [26, 27], and high levels of preoperative anxiety may lead to poor postoperative pain control and increased morbidity [28]. One of the possible mechanisms of preoperative anxiety aggravating postoperative pain is that, GABA participated in the regulation of pathological pain and inhibits hyperalgesia, but anxiety could diminish presynaptic GABA release and impede the action of postsynaptic GABA receptors [29, 30]. This study shows that levels of preoperative anxiety can predict postoperative pain，and the result is consistent with previously reported findings [2, 17, 18]. This means that some patients with preoperative high anxiety may experience severe pain after surgery. In clinical practice we should take some measures, such as injection of sedatives or analgesics before surgery, or non-pharmacologic approaches (including cognitive-behavioral therapy, music therapy, and guided imagery relaxation therapy, etc) to alleviate preoperative anxiety, so as to reduce postoperative pain and discomfort.

CAM-CR scale [20] which had been revised and strictly verified in Chinese was adopted to screen delirium for Chinese patients. Although strong correlation between subjective emotional factors (pain and anxiety) and delirium was generally acknowledged [31], the effect of subjective emotional factors has not been clearly studied so far. In this study, the highest incidence of delirium was the one day after operation, and preoperative high anxiety only had a weak positive correction with postoperative delirium. It is controversial the influence of preoperative anxiety on postoperative delirium. Some studies have shown that preoperative anxiety was a predictor of postoperative delirium [14, 32, 33]. However, the result from most of the studies have revealed that there was no association between preoperative anxiety and postoperative delirium in elderly patients [12–14]. Therefore, more studies about the the correlation between preoperative anxiety and postoperative delirium need to research. In this study, the incidence of postoperative delirium was 25.8%, consistent with the results of previous studies [34]. 24.2% patients had symptoms of delirium on the first day after surgery, and 4.2% patients were still diagnosed as delirium on the third day after surgery. Previous studies have shown that advancing age (> 65 years) is a one of nonmodifiable risk factors for delirium [35], due to elderly patients are more likely to have malnutrition, weakness, preoperative cognitive dysfunction, and brain organic lesions (such as cerebral infarction, microchanges in white matter).

As an acute response after surgical stimulation, postoperative pain often induces psychological, physiological, and behavioral changes such as anxiety, fear, depression, and sleep disorders in elderly patients due to the occurrence or aggravation of their underlying diseases. Two observational studies had found that preoperative pain and depression were associated with an increased risk of delirium [36, 37]. High levels of postoperative pain and using high opioid doses increased the risk for postoperative delirium [16]. however, evidences about the influence of postoperative pain on postoperative delirium are currently limited. In this study, postoperative high pain was considered to be a predictor of postoperative delirium in elderly patients receiving gastrointestinal cancer surgery. Despite the lack of a recognized mechanism of action, the outcome requires more research.

### Potential limitations

There were several limitations in this study. First, due to the workload and patient cooperation, the evaluation time of our scale was an interval of one to two day, which may cause some missed diagnoses. There should be more patients with high pain and delirium. It has been reported that the incidence of delirium in the first two days after surgery is the highest. Delayed delirium can be found on the 7th day after the operation. Second, this study did not include the operation conditions (such as bleeding and fluid volume, the amount of anesthetic) and the use of analgesia pumps that may affect the incidence of postoperative pain and delirium. Third, the scale used for delirium in this study CAM-CR scale, which was revised by Chinese scholar Juan Li [20] based on the CAM scale in 2003. It conforms to the evaluation of Chinese adult delirium, and has high sensitivity and specificity.

## Conclusions

Elderly patients undergoing gastrointestinal tumor surgery had the high incidence of high anxiety before surgery, high anxiety pain and delirium on the first postoperative day. Preoperative high anxiety was positively associated with postoperative high pain and postoperative delirium; and postoperative high pain was positively associated with postoperative delirium. Types of cancer and surgical approaches were as influencing factors of preoperative high anxiety; preoperative high anxiety was an influencing factor of postoperative high pain; and age and preoperative high anxiety were influencing factors of postoperative delirium.

## Supplementary Information

**Additional file 1.**

## Data Availability

All data generated or analysed during this study are included in this published article and its supplementary information files.
